# EEG as a translational biomarker and outcome measure in fragile X syndrome

**DOI:** 10.1038/s41398-022-01796-2

**Published:** 2022-01-24

**Authors:** Aisling Kenny, Damien Wright, Andrew C. Stanfield

**Affiliations:** Patrick Wild Centre, Division of Psychiatry, Kennedy Tower, Royal Edinburgh Hospital, University of Edinburgh, EH10 5HF Edinburgh, UK

**Keywords:** Predictive markers, Predictive markers

## Abstract

Targeted treatments for fragile X syndrome (FXS) have frequently failed to show efficacy in clinical testing, despite success at the preclinical stages. This has highlighted the need for more effective translational outcome measures. EEG differences observed in FXS, including exaggerated N1 ERP amplitudes, increased resting gamma power and reduced gamma phase-locking in the sensory cortices, have been suggested as potential biomarkers of the syndrome. These abnormalities are thought to reflect cortical hyper excitability resulting from an excitatory (glutamate) and inhibitory (GABAergic) imbalance in FXS, which has been the target of several pharmaceutical remediation studies. EEG differences observed in humans also show similarities to those seen in laboratory models of FXS, which may allow for greater translational equivalence and better predict clinical success of putative therapeutics. There is some evidence from clinical trials showing that treatment related changes in EEG may be associated with clinical improvements, but these require replication and extension to other medications. Although the use of EEG characteristics as biomarkers is still in the early phases, and further research is needed to establish its utility in clinical trials, the current research is promising and signals the emergence of an effective translational biomarker.

## Introduction

### Fragile x syndrome

Fragile X syndrome (FXS) is the foremost cause of genetically inherited intellectual disability, estimated to occur in 1 in 4000 males and 1 in 8 000 females [1]. It is caused by a genetic mutation within the X-linked FMR1 gene, arising from a CGG-trinucleotide repeat expansion, and resulting in a deficit of the mRNA binding protein, fragile x mental retardation protein (FMRP). The condition is highly heterogeneous and clinical manifestations vary considerably with mutation extent. Given the X-linked nature of the mutation, males also tend to have stronger clinical presentations than females. Full mutations of greater than 200 repeat expansions regularly result in intellectual disability (ID), heightened anxiety [2, 3] attentional problems and hyperactivity [4–6], disordered sleep [7, 8] and seizures [9]. Several autism spectrum disorder (ASD) characteristics such as perseveration, social anxiety, repetitive behaviours and sensory hypersensitivity are also frequently reported [10–12], with full ASD comorbidity present in about 30-50% of those with FXS [13, 14].

### The Fmr1 KO rodent

Murine models of FXS have driven much of the underlying biological understanding of FXS and have greatly advanced this field of study. The most prominent pre-clinical model, the fragile x mental retardation 1 knock-out (Fmr1 KO) mouse [15, 16], shares several biological and phenotypic similarities with FXS. These mice show abnormal dendritic spine development [17–19], cognitive impairment [20, 21] and behavioural correlates including social impairments, hyperactivity, repetitive behaviours, sensory hypersensitivity, attentional difficulties and susceptibility to audiogenic seizures [22–25]. These findings of parallels between FXS animal models and their human counterparts have paved the way for the development of targeted drug treatments which alter the underlying pathophysiology of the condition. And indeed, at the preclinical stages, there have been several successful trials of targeted FXS treatments which show great remedial promise.

### The clinical pipeline problem

To date, however, only a very limited number of these successes have transferred to the clinical stage. There exists a need to find a single measure which can be observed across humans and rodents; a marker of the condition which can be altered in preclinical trials, and act as better predictor of the likelihood of success when moving to human trials. The heterogeneous nature of FXS results in a range of symptomology across multiple behavioural and cognitive domains, which also means that choosing an appropriate outcome measure in clinical trials is problematic. Debate persists over the cognitive and behavioural measures most suitable to signal successful modification of the underlying biology [26], and the lack of coherence across research means that study results are often difficult to compare.

Recently, there has been growing evidence for the possibility of electrophysiological biomarkers in filling this gap. Research has identified certain characteristic EEG profiles of FXS patients, which tap into the underlying biology of the condition and may indicate potential targets for pharmaceutical alleviation.

The aim of this narrative review is to look at how EEG might be used along the entire pharmaceutical testing pipeline, from rodent model through to clinical trial outcome measure. Finding a single measure which can both indicate clinical targets and treatment success, across human and laboratory models, may allow for greater coherence between research stages and improve success at clinical trial level.

### EEG in FXS: ERP components

EEG abnormalities were first observed in FXS patients through atypical event related potential (ERP) responses. ERP’s are small voltage changes generated in response to specific motor, sensory or cognitive events [27]. Early components (occurring at around 100 ms after onset of event) have been associated with sensory detection and are modality specific, whereas later components typically reflect higher level cognitive processes. ERP’s are most often investigated in FXS populations using an auditory oddball paradigm, in which participants listen to a stream of identical standard tones, randomly interspersed by a deviant or ‘odd’ tone. Such a paradigm was used for the first study of EEG abnormalities in FXS by St. Clair and colleagues [28] to investigate the auditory P3 response. This component occurs as a positive deflection around 300 ms after the presentation of an unexpected stimulus; in this case, a deviant tone. In this study however, both the standard and deviant tones elicited a P3 response in FXS participants and the deviant P3 response demonstrated reduced amplitude and a later latency when compared to healthy controls. This was the first evidence that abnormal ERP’s might exist in those with FXS.

Atypical responses have also been observed in the P2 and N2 ERP components in response to auditory oddball paradigms. But results have been somewhat inconsistent (see Table 1). Some studies have found increased P2 amplitudes in FXS participants in response to standard and deviant tones in the auditory oddball paradigm [29, 30], but not consistently [31, 32]. Similarly, some studies show reduced N2 amplitudes compared to controls [31], and in others, N2 amplitudes were heightened in FXS [32]. ERP’s have also been investigated in the visual modality, though not as frequently and with less consistent results. Findings have included increased amplitudes for visual N70 and N2 [33], and reduced amplitudes for P100 and P3b ERP components [32, 34]. Rigoulot and colleagues [35] also investigated visual components by looking at repetition suppression to face stimuli in FXS patients. The presentation of faces typically elicits a negative deflection around 170 ms after stimulus presentation (the N170 ERP [36]) and with stimulus repetition, a dampening of this neural specific response is commonly observed due to habituation [37]. In this study, conversely, FXS participants showed an elevated N170 response on subsequent presentation of the face stimulus, indicating a differing neural response pattern.

**Table 1:** Summary of ERP differences in humans with FXS.

Authors	Comparison group (*N*)	Method	Results
Castrén et al. [38]	Fragile X syndrome (5) Neurotypical controls (4)	Auditory oddball	↑N1 amplitudes ↓N2 amplitudes
Côté et al. [34]	Fragile X syndrome (14) Tuberous sclerosis complex (9) Down syndrome (19) SYNGAP1-related ID (8) Neurotypical controls (55)	Auditory habituation and oddball	Larger P1-N1 and N1-P2 peak-to-peak values in FXS. Stronger peak-to peak repetition suppression in FXS compared to neurotypical controls.
Ethridge et al. [31]	Fragile X syndrome (14) Neurotypical controls (15)	Modified auditory gating task	↓N1 habituation ↓N2 amplitudes
Ethridge et al. [29]	Fragile X syndrome (38) Neurotypical controls (40)	Auditory habituation task	↑N1 amplitudes ↑P2 amplitudes However habituation patterns were retained for both N1 and P2
Ethridge et al. [46]	Fragile X syndrome (41) Age matched neurotypical controls (27)	Auditory oddball	↑N1 amplitudes ↑P2 amplitudes
Knoth et al. [33]	Fragile X syndrome (12) Chronological age-matched neurotypical controls (12) Developmental age-matched neurotypical controls (9)	Auditory and visual evoked paradigm	Auditory: ↑N1 amplitudes ↑P2 amplitudes ↑N2 amplitudes ↑N2 latencies Visual: ↑N70 amplitudes ↑N2 amplitudes
Rigoulot et al. [35]	Fragile X syndrome (13) Neurotypical controls (24)	Visual habituation	↑N170 amplitudes to second presentation of stimulus
Schneider et al. [43]	Fragile X syndrome (12) Neurotypical controls (40)	Auditory oddball	↑N1 amplitudes ↑P2 amplitudes
St Clair et al. [28]	Fragile X syndrome (33) Downs syndrome (90) Neurotypical controls (83)	Auditory oddball	↑N1 amplitudes ↑P2 amplitudes ↓P3 amplitudes
Van der Molen et al. [32]	Fragile X syndrome (11) Neurotypical controls (22)	Visual and auditory oddball	Findings from both tasks: ↑N1 amplitudes ↑N2b amplitudes ↑N2b latencies ↓P3b amplitudes ↑P3b latencies
Van der Molen et al. [30]	Fragile X syndrome (16) Neurotypical controls (20)	Auditory oddball	↑N1 amplitudes ↑N2b amplitudes ↑P2 amplitudes ↑N2b latencies ↓P3a amplitudes ↑P3a latencies

While these ERP findings demonstrate modified neural activity in FXS, the results are inconsistent, and little is currently known about the exact relationship between the ERP alterations presented thus far and FXS biology. Therefore, the utility of such ERP’s as potential biomarkers is uncertain.

**Fig. 1:**
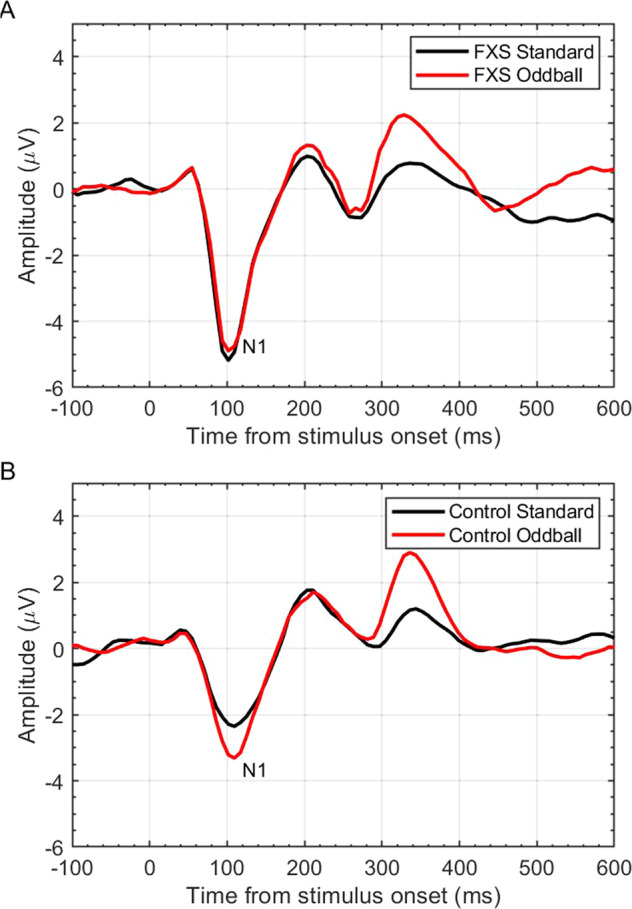
Example of N1 ERP in response to standard and deviant tone presentation in auditory oddball task for FXS and for controls. **A** The exaggerated N1 amplitude (reported in microvolts (μV), across time (ms)) response to both standard and oddball tones as observed in FXS. **B** The neurotypical control response; exaggerated N1 amplitude to oddball tone, but attenuated N1 amplitude to the repeated standard tone.

The possibility of using EEG related biomarkers for targeted treatments in FXS became more feasible with findings of a regularly observed atypical ERP response. First found to be significantly different from controls in St. Clair’s study, the abnormal auditory N1 response has been one of the most consistently observed ERP abnormalities in FXS. This component typically shows a negative deflection peak around 100 ms after the onset of an auditory stimulus and has been linked in FXS to cortical hyper excitability, which might directly relate to the underlying pathophysiology of the condition. In the auditory oddball paradigm, deviant tones typically elicit significantly larger N1 amplitudes in healthy controls compared to standard tones. This deviant specific response is absent in patients with FXS, for whom N1 amplitudes are increased more than controls to both standard and deviant stimuli in both auditory [30, 33, 38] and visual modalities [32] (see Fig. 1). The auditory N1 has been associated with states of arousal [39] and enhanced N1 activation in patients with FXS signals auditory hypersensitivity, or an overactive response to auditory stimuli. Heightened sensory sensitivity and hyper arousal have long been observed in FXS and have been implicated in core issues with inattention, hyperactivity, anxiety, social avoidance, and sensory defensiveness [32, 40–42]. In addition, habituation of the N1 response is also regularly found to be reduced or absent in FXS patients compared to controls when presented with a string of identical tones [31, 32, 43]. Neuronal habituation is considered an early and fundamental form of learning [44], and impairments in habituation are a common feature found in other forms of ID. As such, attenuated neuronal adaptation has been suggested to contribute to core learning deficits in FXS [45], although more recent studies have found N1 habituation to be heightened in FXS [34, 46]. So how exactly this habituation marker relates to FXS symptomology is uncertain. For increased N1 amplitudes, however, there seems to be greater evidence for its connection to underlying biology.

### Cortical hyper excitability, glutamate and GABA

This elevated electro cortical response to auditory stimuli seen in the N1 component is proposed to reflect cortical hyper excitability, a concept which had already been hypothesized based on the glutamatergic/GABAergic excitatory imbalances in FXS. As the main inhibitory neurotransmitter in the brain, GABA exerts inhibitory action through GABAa ionotropic neurotransmitter receptors and GABAb metabotropic receptors [47]. Studies in Fmr1 mouse models have shown altered GABA receptor subunit expression [48] and reduced GABA production [49, 50], suggesting decreased GABA-related inhibition in FXS. Glutamatergic activity, conversely, is elevated in FXS. The mGluR (metabotropic glutamate receptor) theory of FXS [51] was developed based on findings of elevated mGluR-dependent long-term depression (LTD) in hippocampal regions of KO mice, which requires mGluR5 activation [52]. FMRP ordinarily functions as an inhibitor of mGluR-dependent protein synthesis and, consequently, mGluR-dependent LTD. As such, deficits in FMRP result in exaggerated mGluR5 related protein synthesis and LTD, leading to a propensity for heightened excitatory activity, exacerbated by simultaneous inhibitory deficits. This imbalance of glutamatergic/GABAergic neurotransmitter pathways has been thought to underlie many of the phenotypic characteristics in FXS and has been the focus of several pharmaceutical remediation studies [53–55]. It is proposed that the exaggerated electrophysiological characteristics observed in FXS relate to this over-excited neural system and reflect underlying biology. Findings that EEG related characteristics might reflect aberrant excitatory and inhibitory activity signalled its potential as a translational biomarker.

### Spectral abnormalities and functional connectivity

ERP’s are not the only electrophysiological differences observed in FXS. Certain frequency bands of neural oscillations have also been implicated in FXS symptomatology. Neural oscillations are rhythmic patterns of neuronal activity, which can occur at the individual neuronal level, or as a larger level synchronization across multiple neurons. This combined oscillatory activity is recorded as the EEG signal and can occur across a spectrum of frequency bands. These bands are typically broken into delta (1–3 Hz), theta (4–8 Hz), alpha (8–12 Hz), beta (13–30 Hz) and gamma (30–100 Hz).

Time-frequency analysis of the EEG signal, which provides information about the change in frequency information over time, has shown a relation between irregularities in these frequency spectrums and cortical hyper excitability, particularly in the gamma frequency bands. Gamma activity has been associated with several cognitive and perceptual processes including memory, attention, learning and perception [56], and abnormalities in this frequency range have been observed in a host of neuropathological disorders, such as schizophrenia, Alzheimer’s disease, ADHD and ASD [57–60]. Gamma band activity is sometimes sub-divided into high and low gamma bands (or low/mid and high/fast [61]). The ranges taken to signify high gamma activity varies, but generally includes those frequencies above 60–80 Hz [62, 63]. What distinct functional role high gamma plays is uncertain, but could provide an additional avenue of inter-cortical communication [64], a role which gamma band activity more generally is hypothesized to play [65]. Although many of the studies included here do not subdivide gamma bands, it is worth noting that these gamma band divisions may arise from differing mechanisms [63, 66].

Excessive high frequency resting state and evoked (in response to stimulus/task) gamma have been reported in ASD in relation to auditory stimuli [67, 68] and associated with degree of developmental delay in boys with autism [69]. Ethridge and colleagues [31] similarly demonstrated spectral abnormalities in gamma band activity for participants with FXS when exposed to auditory stimuli. In this study, individuals with FXS and age matched controls listened to a series of identical 1000 Hz tones. Time-frequency analyses exhibited an increase in both stimulus-specific and non-specific single trial gamma power alongside decreased stimulus invoked gamma phase locking in FXS patients compared to controls [31]. Typically, gamma band activity is expected to synchronize or ‘phase-lock’ to high frequency stimulus input, and with habituation to repeated stimuli, increased gamma synchronization should be observed [70]. Instead, these results show elevated gamma activity in those with FXS, which hinders subsequent synchronization of gamma activity to stimulus presentation. Reduced gamma spike phase locking was associated with exaggerated N1 amplitudes, and increased task relevant gamma power was related to the reduced capability to attenuate this response through stimulus repetition. The increased background gamma power, and the inability to successfully synchronize gamma activity to the stimulus, are suggested to signal both a hyper excitable and disorganized system.

Similar findings were repeated by Ethridge and colleagues [71] who examined neural synchronization using a ‘chirp’ stimulus; a 1000 Hz tone presentation, amplitude modulated by a sinusoid which increased linearly in frequency from 0 to 100 Hz, which drives synchronous oscillatory activity. Again, FXS participants showed attenuated synchronized gamma band activity to stimulus presentation, a deficit which was highly correlated with increased non-specific gamma power. This association between heightened local neural network excitation and attenuation of synchronized activity at the level of neuronal populations in sensory circuits, was specific for gamma activity. In addition, elevated levels of single trial gamma power correlated with increased parental reports of sensory hypersensitivity and autism related social impairment, a finding replicated elsewhere [72]. Both studies suggest that increased levels of non-specific gamma activity act as background ‘noise’, due to the hyper excitability of the system, which then hinders the ability to synchronize gamma frequency to the presentation of a high frequency stimulus.

Similar results were again replicated in very recent studies by Ethridge and colleagues: (1) using auditory chirp and auditory habituation tasks (finding increased N1 amplitudes, reduced gamma phase-locking and increased gamma frequency power) [29] and (2) using an auditory oddball paradigm (resulting in increased N1 amplitudes and gamma power) [46]. The latter study conducted a retest analysis on 14 of the younger participants one month following initial testing and found high retest reliability of N1 amplitude and gamma power responses. They also found the developmental trajectory of these responses to be similar in both FXS and control groups. Findings of developmental similarity and retest reliability of these EEG characteristics amongst younger participants, indicate they may be strong candidates for biomarker investigation in studies with children. Interestingly, however, this most recent study by Ethridge and colleagues [46] also found greater sensory avoidance (based on parent reported scores from the Sensory Profile 2) to be correlated with lower gamma power, contradictory to earlier findings [31, 71]. The authors suggest this might be due to previous work associating higher gamma power with auditory processing specifically, whereas this study took measures of sensory experiences more generally, which was then correlated with lower gamma power.

Spectral abnormalities have also been reported in other frequency bands (see Table 2). Resting state studies of FXS have demonstrated increased resting theta power and reduced upper alpha power across several neural areas compared with neurotypical controls, suggesting globally affected circuitry in FXS [29, 73]. Failure to show repetition suppression effects in response to visual stimuli has been observed across frontal and parietal-occipital areas in the theta band in FXS patients. [35] Aberrant functional connectivity has also been observed, with both long- and short-range connectivity found to be reduced for alpha band activity, but increased for theta oscillations [74]. Wang and colleagues [72] similarly found diminished long-range connectivity in alpha, as well as beta bands. Short-range gamma band connectivity was enhanced, and although increased theta connectivity was not replicated, they found increased theta-gamma coupling. Augmented connectivity across more distal electrode sites indicates a wider spread of gamma activity, or cortical excitability, as previously elucidated through increased relative gamma power. This, alongside reduced long-range alpha and beta connectivity, has interesting possible implications. Low frequency oscillations have been shown to be involved in top-down inhibition and modulatory control in more globally distributed networks [75], whereas high frequency oscillatory activity such as gamma has been associated with more local neural network activity. More widespread gamma connectivity in FXS may constitute a deficit in top-downregulation resulting from reduced alpha power and long-range connectivity and attenuated alpha-gamma coupling. In Wang’s study [72], all FXS participants showed abnormal long-range functional connectivity. In addition, a recent study by Schmitt et al. examining the neural signatures involved in speech production found reduced pre-speech low frequency fronto-temporal coherence, but increased gamma power in frontal areas in FXS compared to typically developing controls. These alterations were related to greater issues in measures of speech production. Further, in TDC’s, elevated gamma power was correlated with increased fronto-temporal coherence, a relationship which was not observed in FXS. It is of note for future research to elucidate further this cortico-cortical functional connectivity and its role in increased gamma power and neuronal hyper excitability.

**Table 2:** EEG spectral power findings in FXS.

Authors	Comparison group (*N*)	Method	Results
Ethridge et al. [31]	Fragile X syndrome (14) Neurotypical controls (15)	Modified auditory gating	Increased gamma power Decreased gamma phase-locking
Ethridge et al. [29]	Fragile X syndrome (38) Neurotypical controls (40)	Auditory chirp	Increased gamma and theta power Decreased upper alpha power Decreased gamma phase-locking
Ethridge et al. [46]	Fragile X syndrome (41) Neurotypical controls (27)	Passive auditory oddball	Increased gamma power
Rigoulot et al. [35]	Fragile X syndrome (13) Neurotypical controls (24)	Visual habituation	Weaker repetition suppression in FXS
Van der Molen et al. [73]	Fragile X syndrome (8) Neurotypical controls (12)	Resting State	Increased theta power Decreased upper alpha power
Van der Molen et al. [74]	Fragile X syndrome (8) Neurotypical controls (12)	Resting State	Decreased global functional connectivity for upper alpha and beta Increased connectivity for theta (fronto-posterior; frontal-frontal; posterior-posterior)
Wang et al. [72]	Fragile X syndrome (21) Neurotypical controls (21)	Resting State	Increased gamma power Increased spatial spreading of phase-synchronized gamma activity Increased negative theta-to-gamma band amplitude coupling Decreased alpha-to-gamma band amplitude coupling.

### Gamma activity and FXS biology

The exact underlying mechanisms generating gamma oscillatory activity are still a matter of debate. However, there are currently two theoretical models thought to be responsible for gamma oscillations in local circuits: the inhibitory–inhibitory (I–I) and excitatory–inhibitory (E–I) models [76]. Both models are in fact expected to be at work in the brain, with the model employed likely dependent on the brain area involved. The I–I model suggests gamma oscillations emerge through GABA receptor mediated interneuron activity under conditions of tonic excitation [77], whereas the E–I model is thought to generate gamma oscillatory activity through excitatory pyramidal cell and inhibitory interneuron interactions [78]. Both cases underline the significance of inhibitory GABAergic interneurons in the emergence of gamma oscillations [79–81]. Low threshold spiking (LTS) interneurons have been shown to contribute to neural network synchronization in various frequencies including gamma [82] and fast-spiking (FS) interneuron networks have been implicated in the synchronization of gamma oscillatory activity through the coordinated activity of inhibitory post-synaptic potentials (IPSP’s) onto excitatory neurons [83, 84]. These fast-spiking neurons are also preferentially activated in the 40–100 Hz (gamma) frequency range [79, 85]. Laboratory models of FXS have shown decreased density and local neocortical excitation of FS interneurons [86, 87] and reduced activation of LTS interneurons [88] in Fmr1 KO mice. This may result in the inability to successfully synchronize gamma oscillatory activity to stimulus presentation, as observed in FXS. Although increased hyper excitability has repeatedly been shown in mouse models of FXS [89], how this leads to increases in gamma power is less well understood. However, there may be a role in reduced inhibitory interneuron activity onto excitatory pyramidal neurons, leading to an exaggerated excitatory response [88]. More research is required to elucidate the exact link between gamma band activity and underlying biology of FXS.

### EEG in rodent models: abnormalities and similarities

Despite variations in brain size and structure, oscillatory temporal frequencies are highly replicable across mammalian species [61]. As such, one of the main potential utilities of EEG related biomarkers is their remarkable similarities in both rodents and humans. Cortical hyper excitability has been demonstrated in animal models of FXS [50, 90–92], which have also shown hypersensitive responses to auditory stimuli [25, 93]. Additionally, Fmr1 KO mice show protracted ‘UP’ state durations for gamma range frequencies [94]. These UP states are short phases of local network activity resulting in regulated states of depolarization and firing synchrony amongst adjacent neurons. Such states arise through the excitatory/inhibitory feedback balance, altered towards increased excitation in the Fmr1 KO mouse and which supports prolonged durations of local network ‘UP’ states [95]. The termination of such UP states has been linked to low-threshold spiking (LTS) interneurons [96] the activation of which, as previously mentioned, is hampered in Fmr1 mice.

These biological abnormalities in Fmr1 KO mice have also given rise to EEG findings similar to those in humans with FXS, including reduced habituation to auditory stimuli [97], increased gamma band activity [89, 98, 99] and reduced synchronization of task relevant gamma [99] (see Table 3). Reduced task-specific gamma synchronization was found by Lovelace and colleagues. [99] using a chirp tone paradigm, similar to that employed in an earlier human study [71]. Using tightly matched paradigms in rodent studies to replicate findings from clinical populations, suggests EEG might allow for tighter equivalence than is currently the case with behavioural paradigms. Lovelace et al. also demonstrated conserved EEG abnormalities when accounting for movement state, which had previously been suggested as a possible contributor to spectral abnormalities observed in Fmr1 KO mice [100]. Increased auditory evoked gamma power has also been tentatively linked to social impairment and working memory in FXS mouse models [100], suggesting potential for these EEG alterations to underlie changes in behaviour. These findings in mouse models have recently been repeated in Fmr1-KO rat models, demonstrating increased gamma power and reduced inter-trial coherence in response to auditory stimuli [101]. This further evidences the potential for EEG abnormalities observed in FXS to transfer across species. Recent advances in murine EEG systems have also led to the possibility of stronger animal-human parallels. A 30-channel multi-electrode array system was recently developed for use in rodents. When tested on Fmr1 KO mice, auditory chirp stimuli elicited remarkably similar EEG phenotypes to those observed in humans, including higher resting state gamma power and increased event related power compared to wild type mice (although increases were observed across various spectral bands), increased auditory N1 amplitude and reduced phase-locking to stimuli in the gamma range [102]. This reduction in inter-trial phase coherence was observed across all brain regions, and was low-gamma specific, leading the authors to suggest the utility of this phase-locking coherence as a reliable translational biomarker.

**Table 3:** EEG findings in the Fmr1 KO rodent model of FXS.

Authors	Method	Result
Berzhanskaya et al. [90]	Resting state	Increased high-frequency power. Reduced low-frequency power. Reduced synchronization.
Jonak et al. [102]	Resting state; auditory chirp train	Increased resting EEG power. Increased auditory ERP amplitudes (P1, N1, P2) Increased event-related power. Reduced inter-trial phase coherence.
Kozono et al. [101]	Auditory click train	Increased gamma power. Reduced alpha and beta power. Reduced gamma synchronization.
Lovelace et al. [97]	Auditory habituation and auditory oddball	Reduced habituation of auditory N1 amplitude.
Lovelace et al. [99]	Auditory chirp train	Increased resting gamma and delta power. Reduced auditory evoked synchronization. Increased background non-phase locked single trial gamma power. Increased N1 amplitudes and longer P1 latency to auditory stimuli.
Lovelace et al. [145]	Auditory chirp and broadband noise trains	Increased resting low gamma power. Increased background single trial low gamma power. Increase in induced non-phase locked gamma power.
Lovelace et al. [162]	Auditory chirp and click trains	Increased resting power across all frequency bands except alpha. Reduced inter-trial phase coherence in beta and gamma bands. Increased sound-induced beta and gamma power
Sinclair et al. [100]	Auditory broadband noise train	Increased baseline and auditory evoked gamma power.
Wen et al. [161]	Auditory broadband noise train; Resting state	Increased baseline gamma power. Increased N1 amplitude in frontal cortex.
Wong et al. [158]	Immobile awake and sleep-like resting states	Increased gamma band power in frontal cortex of male rats during sleep-like state. Reduced low frequency power during immobile, sleep-like state. Reduced theta band power in female rats during awake, immobile state.

### The preclinical to clinical pipeline: issues in current trials

The need for a more effective biomarker can be observed in the gap between successful pharmaceutical trials in laboratory models and failure of the same medications in human studies. Several avenues of pharmaceutical development have been explored based on the current knowledge of the molecular and biological mechanisms underpinning FMRP deficiency in Fmr1 KO mouse models. Most avenues have focused on the activity of group 1 metabotropic glutamate receptors (mGluR) and GABAergic receptors and their respective signalling pathways. Rescue of several FXS features have been observed in KO mice following both genetic downregulation of mGluR5 expression [103, 104] and administration of specific mGluR5 antagonists, which lessen the activity and downstream signalling of mGluR. AFQ056/mavoglurant has shown remediation of dendritic spine dysmorphology and behavioural phenotypes [105–107] in Fmr1 KO mice and similar findings have been shown with MPEP [108–110] and fenobam [111, 112]. Despite this preclinical success, translation to clinical outcomes has been lacking. Several recent, double-blind, placebo-controlled studies found no benefit of mavoglurant administration on several outcome measures of behavioural characteristics in large FXS cohorts [53, 113, 114]. Similar unsuccessful findings have been observed for the mGluR5 negative allosteric modulator, basimglurant [115]. Preclinical studies with GABA receptor agonists have similarly shown an ability to rescue and reduce susceptibility to audiogenic seizures [116], correct synaptic abnormalities [117] and reduce elevated protein synthesis and social behaviour deficits [118]. However, early clinical studies of GABA-ergic compounds showed limited improvements [119]. Phase 3 studies of the selective GABAb receptor agonist arbaclofen (STX209, R-baclofen) showed no improvements in primary social behaviour outcomes which had been found in a previous preliminary study [120] and only limited benefits in other behavioural measures [55].

One issue facing these pharmaceutical trials in FXS is that outcome measures used in laboratory models sometimes lack strong equivalence both to the outcome measures used in human trials and to the targeted behaviour in humans. Although mouse models have recapitulated certain FXS phenotypes, the underlying mechanism driving these behaviours may not be comparable, and inversely, biological similarities may not result in behavioural parallels. For example, anxiety behaviours, so often observed in FXS patients, have yielded mixed results in studies of Fmr1 KO mice [121–123]. This could signal species-specific differences in behavioural presentation or suggest that the paradigms used may not capture the same behaviour. Rodent anxiety tests typically include an open versus closed area, with increased time and exploration in open areas associated with decreased anxiety. The elevated plus maze task, assessing the ratio of time spent in the open versus closed arms of a plus-sign shaped maze, is commonly used as a measure of anxiety in rodents, and particularly as a measure of the remedial capabilities of anti-anxiety drug treatments [124]. Interestingly, Fmr1 KO mouse models have frequently shown decreased anxiety (more time in open/light space), compared to wild type littermates, in these kinds of anxiety tests [121, 125], which is contrary to human findings [2, 3]. It has been suggested this increased open arm exploration is potentially indicative of increased locomotor activity or hyperactivity rather than increased anxiety [125]. Even if this is the case, it only functions to further highlight the difficulty in capturing the targeted translational behaviours effectively. These sorts of conflict tests have also been deemed quite sensitive to variabilities in genetic background strain and age of the rodent as well as housing and testing facilities [126].

Despite the issues with behavioural equivalence in preclinical outcome measures, their strength lies in the ability to track pharmaceutical alterations at this preclinical stage. Conversely, in human trials, though outcome measures may capture the phenotype of interest successfully, their ability to capture treatment related changes is less compelling. Most measures employed in clinical trials have been deemed moderately effective as outcome tools and there exists limited data on their sensitivity to detect pharmaceutically driven changes [26]. The majority of outcome measures involve parent/caregiver reports, which are inherently susceptible to scoring variability and placebo effects [127]. The most widely used primary outcome measure for clinical trials in FXS has been the Aberrant Behaviour Checklist- Community (ABC-C), a parent or caregiver report which assesses maladaptive behaviours as perceived in the weeks prior to rating. Subscales aim to target behaviours relating to irritability, hyperactivity, stereotypic behaviour, inappropriate speech, and social withdrawal. This scale has recently been adapted for FXS (ABC-CFX), adding better representative subscales of autistic-like behaviours and social anxiety [128]. Although it has been proposed to have good psychometric properties, limited data exists on its sensitivity to treatment response [26, 129]. The ABC-C has detected treatment changes in some previous studies, but often only on specific subscales, and not consistently [55, 120, 130, 131]. This trend towards inconsistency has been observed in other common measures including the Social Responsiveness Scale (measuring social impairment), the Clinical Global Impression, severity and improvement scales (CGI-S, CGI-I) (measuring symptom severity or improvement respectively) and the Visual Analogue Scale (VAS), which measures a behaviour predefined by parents [114, 115, 132, 133]. The primary and secondary outcome measures used in clinical trials varies widely between studies, often making comparisons between studies difficult. Whether these measures successfully reflect a change in underlying biological mechanisms is also uncertain and the lack of a unified measure that can track biological remediation across both animal and human studies remains an issue in the translational pipeline.

### EEG as an outcome measure

EEG presents the possibility of a coherent outcome measure between humans and lab models of FXS, with similar testing paradigms and a more closely matched quantifier of treatment efficacy. It has begun to make its way into preclinical drug studies and has shown some promise as a measure of preclinical success. Racemic baclofen, a GABA-B agonist, rescued elevated auditory invoked gamma power in Fmr1 KO mice [100]. Similarly, acamprosate (an NMDA receptor antagonist and GABA-A positive allosteric modulator) successfully reduced the prolonged ‘UP’ states observed in FXS mouse models [134]. More recently, the PDE10A inhibitor TAK-063 was tested in the Fmr1 KO mouse model and resulted in significant increases in inter-trial phase coherence across several brain regions, in response to an auditory chirp stimulus [135]. PDE, or phosphodiesterase, is an enzyme that breaks down cyclic AMP, an intracellular signalling molecule whose production is reduced in FXS [136]. Although another PDE targeting compound has just completed phase 2 clinical trials in patients with FXS, the use of EEG as an outcome measure for the effectiveness of these drugs in humans has not yet been fully explored.

Tentative evidence for the possibility of EEG to create a cohesive translational pipeline has been observed in the case of minocycline. Minocycline is a tetracycline analogue shown to recover structural synaptic abnormalities and spine dysmorphology in Fmr1 KO mice [137] with observable behavioural alterations. More commonly prescribed for its antibiotic properties, the benefits of minocycline in FXS are thought to be enacted through the inhibition of matrix metalloproteinase-9 (MMP9) [138], a locally translated protein, regulated by FMRP and overexpressed in FXS [137, 139]. Studies investigating minocycline in the KO mouse model have observed wide ranging behavioural remediation, including increased ultrasonic vocalizations in males [140, 141] improvements in memory recognition of social [142] and novel objects [143], and reductions in locomotor activity, susceptibility to audiogenic seizures and perseverative behaviour [144]. Additionally, the Fmr1 KO mouse model has been observed to have increased MMP-9 gelatinase activity and elevated resting gamma power [145], suggesting a possible connection between MMP9 and gamma activity. More recently, Lovelace et al. [162]. investigated the effects of a 10-day course of minocycline treatment on EEG abnormalities in Fmr1 KO mice. Specifically, whether sound-evoked or resting state EEG measurement better tracked minocycline related changes. They used a “chirp” paradigm similar to those mentioned previously, and a click train paradigm to drive auditory steady state responses (ASSR) at a frequency of 40 Hz. Minocycline rescued the EEG characteristics in Fmr1 KO treated mice compared to vehicle-treatment including (1) reduced inter-trial phase coherence (ITPC) across beta-gamma frequencies in the auditory cortex in response to the chirp paradigm; (2) reduced ITPC for 40 Hz ASSR in the frontal cortex; and (3) increased on-going power in delta and gamma (60–80 Hz) 100-400 ms following stimulus presentation. They also observed a shift from high to low gamma power in the auditory cortex in Fmr1 KO minocycline-treated mice compared to vehicle-treated WT mice. Although minocycline did reduce resting gamma power, this was also observed with vehicle treatment, suggesting that resting state measures may not track minocycline-specific changes as accurately as sound-evoked measurements.

Clinical trials in FXS patients have shown similar beneficial effects of minocycline treatment on behavioural outcomes. A pilot survey study conducted by Utari and colleagues [146] reviewed minocycline effects in 50 individuals with FXS. Parents reported improvements in language, attention, and social communication as well as reduced anxiety. Behavioural improvements were also reported in irritability, stereotypy, hyperactivity, and inappropriate speech subscales of the aberrant behaviour checklist (ABC-C) in a separate open-label study [147]. These authors also reported statistically significant improvements in clinical global impressions scales (CGI-S, CGI-I) and the mitigation of several parent-defined behaviours using a Visual Analogue Scale (VAS); findings which were replicated in a later, randomized, double-blind, placebo-controlled crossover trial [133]. These results are promising but require replication. One issue inherent in these previous investigations was the over reliance on parental reports of improvement, introducing bias into necessarily objective research. The effects of minocycline on electro cortical activity in FXS were investigated in a pilot study [43] on a subset of children from a larger, double-blind crossover trial [133]. A passive auditory oddball paradigm was used to investigate auditory ERP amplitudes. Results showed significant attenuation of auditory N1 amplitude and increased habituation following 3 months of minocycline treatment. The electro cortical changes observed following administration of a drug which improves behavioural and clinical outcomes in patients with FXS supports the feasibility of EEG as a sensitive clinical outcome marker. These authors did not investigate spectral abnormalities, however, previous links have correlated elevated N1 amplitude and increased gamma power. The observed benefits of minocycline on FXS ERP responses and behavioural outcomes demonstrates the possibility of EEG to act as an effective measure of pharmacological success, both in rodents and humans, and signals the future potential of EEG as a translational biomarker in FXS research.

This potential has been supported by endeavours to develop biomarkers in other psychiatric conditions and neurodevelopmental disorders. For example, investigations of EEG biomarkers have been conducted in Alzheimer’s [148], major depressive disorder (MDD) and bipolar disorder (BD) [149], psychosis [150], neurodevelopmental disorders such as ADHD [151], ASD [152] and other syndromic forms of autism (e.g. Rett’s syndrome [153]). While many of these are still in the investigative stages, with the need for more research, including larger sample sizes and cross site reproducibility, much can still be learned from the attempts at biomarker development in other conditions. Recently, the United States Food and Drug Administration (FDA) and the European Medicines Agency (EMA) have given support for the use of the N170 ERP as a prognostic marker in clinical trials of ASD patients without intellectual disability. This is the first EEG related biomarker accepted for use in a psychiatric disorder. The N170, which is associated with early stages of face processing, shows longer latencies to face stimuli in participants with ASD when compared to controls [154] and potentially contributes to the social deficits observed in ASD, a core symptom of the condition. Like FXS, ASD is a very heterogeneous disorder. As such, the ability to stratify patients into more homogenous groups should aid the development of more targeted drug treatments. Investigation into the N170 ERP in ASD involved large multicentre studies across Europe (EU-AIMS Longitudinal European Autism Project; LEAP) [155] and the US (Autism Biomarkers Consortium for Clinical Trials; ABC-CT [156]. To improve collection across multiple sites, these investigations have also produced transparent and well-documented research procedures [153] enabling tighter reproducibility across locations, features that are currently lacking in FXS biomarker research, but which are needed for future clinical trials.

## Discussion

EEG is a non-invasive, easy to use technology that is generally well-tolerated in populations with neurodevelopmental disorders, and easy to replicate [29, 157]. Recent interest in the feasibility of EEG as a translational biomarker and sensitive outcome measure has been propelled by its potential ability to act as an intermediary between underlying biological pathophysiology and measures of clinical improvement. One of the major issues for treatment development in FXS, is that success observed in preclinical trials fails to exhibit similar benefits when trialled at the clinical level. But the similarity in EEG paradigms used and abnormalities elucidated between rodents and humans allows for greater translational equivalence than has previously been seen with behavioural outcome measures. This may lead to more efficient detection of rodent-tested drugs, which are likely to succeed in human trials and allow for a more sensitive measure of biological changes when tested clinically. In addition, Ethridge and colleagues [29] repeated their abnormal EEG findings at a different collection site, with a new patient cohort, using a different EEG system, and with multiple researchers contributing to the data processing phases. This shows promise for the robustness of these electro cortical findings and the potential for more wide-scale and accessible use of EEG, particularly in neurodevelopmentally atypical populations, where flexibility in data collection is often required.

While these initial findings are encouraging, there remains a paucity of EEG research in FXS, and some lingering uncertainty as to the exact link between these EEG characteristics and both biological mechanisms and clinical outcomes. Although there have been some exploratory correlations found between EEG abnormalities and clinical outcome measures in FXS, results have been mixed. Heightened N1 amplitudes have been correlated with greater abnormalities on the Sensory Profile questionnaire [31], while increased single-trial gamma power and decreased phase-locking to auditory chirp stimulus have been significantly correlated both with sensory hypersensitivity (based on scores from the Adolescent and Adult Sensory Profile) and autism spectrum behaviours (based on scores from the Social Communication Questionnaire; SCQ) [71, 72]. However, contrary to these findings, Ethridge and colleagues [29] found that increased N1 amplitudes in males was associated with decreased SCQ scores, suggesting less autism related behaviours, and elsewhere, increased sensory sensitivity and avoidance was associated with lower relative gamma power in FXS [46]. Though there does appear to be a link between these EEG characteristics and sensory sensitivities and autism related behaviours, how exactly they relate is less clear. Further work is needed to clarify the relationship between behavioural outcomes and EEG abnormalities.

There are, in fact, several areas that could benefit from further investigation in the future. The correlation between severity of gamma power increases and increased cognitive deficits signals potential for EEG markers in the future to pinpoint those who may benefit most from specific treatments, allowing for more tailored clinical trials and better participant retention. Indeed, the glutamatergic antagonist mavoglurant has previously shown beneficial effects when patients were subdivided based on molecular stratification, despite no significant pre-stratification findings [132]. Further research should aim to clarify the link between EEG abnormalities and clinical correlates, and if these may be used for patient stratification. In addition to this, we have scant knowledge of gender-based differences in electrophysiological presentation and clinical response. Ethridge’s 2019 study [29] found increased N1 amplitudes had differential clinical correlations based on gender (although this may be related to differences in severity of intellectual disability). A recent study in the rat FXS Fmr1 KO model found some differential EEG profiles based on gender, with males, but not females, showing increased gamma band activity during sleep-like state, and observations of reduced theta in females only during awake state [158]. In any case, we have insufficient information about the differences in EEG characteristics between males and females affected by the disorder and how this could translate to differential clinical responses. Should EEG characteristics be used as biomarkers in the future, this is one potential area to which their utility can be extended.

Finally, how these EEG characteristics change across development, and particularly in relation to changes in biological mechanisms underlying the FXS phenotype, is still uncertain [159]. N1 amplitudes have been shown to be affected by age [29, 160]. Less is known about auditory cortical gamma activity, though evidence exists for age -dependent genotypic differences in stimulus evoked gamma band power [161]. However, Ethridge and colleagues [46] recently found developmental trajectories of P1 and N1 amplitudes, P2 latency and increased gamma power to auditory stimuli replicated that of controls. This suggests that although these responses are abnormal, they follow a typical course of development (except for P2 amplitude). This, alongside retest reliability in younger children, strengthens the utility of EEG measures in clinical trials in younger populations with FXS. Further investigation is still required, however, to understand how electro cortical activity changes over time, if there are ages at which the attenuation of EEG abnormalities is most likely, and, therefore, treatment most effective.

## Conclusion

Though knowledge of precisely how the Fmr1 mutation affects electro cortical functioning is still under scrutiny, the changes which have been observed thus far justify further research given their possible utility in drug development. The outcome measures and biomarkers which have been employed in previous studies have led to very few tangible clinical results, and translation to a widely available targeted drug has rarely transpired. The possibility of EEG markers bridging that gap, although preliminary, appears promising.
